# Reviewers in 2020

**DOI:** 10.1098/rsos.210300

**Published:** 2021-04-07

**Authors:** Jeremy K. M. Sanders

**Affiliations:** Editor-in-Chief

To say that 2020 was a difficult year is perhaps an understatement. Lockdowns, virtual and remote working, homeschooling, and the many individual tragedies for which COVID has been responsible have made it exceptionally hard for the vast majority to operate ‘normally’. It is with heartfelt thanks then that the editors and administrative office of *Royal Society Open Science* would like to thank all reviewers who contributed their time and expertise by refereeing manuscripts submitted throughout 2020—we know it has not been easy.

As we have done for several years now, to provide an opportunity for recognition, we list the names of all reviewers who have kindly contributed to the journal (unless they have opted out). This article is made permanent and citeable by its digital object identifier (DOI). We encourage you to quote your contribution in your applications for tenure, promotion and grants, or other forms of assessment. Where you have provided it, we have included your ORCID, so your contribution can be unambiguously assigned to you.

The team greatly appreciate all the work our reviewers do on behalf of the journal, and we look forward to working with you again in 2021. We hope it will be an easier year.

Abbas F
Abbas SN
Abbasi Q
Abdessemed A https://orcid.org/0000-0001-5190-5991
Abdul Rahman F https://orcid.org/0000-0001-6189-3365
Abdullah Z
Aboelalla M https://orcid.org/0000-0002-8579-0093
Abouelmaaty T https://orcid.org/0000-0002-4877-0085
Aczel B https://orcid.org/0000-0001-9364-4988
Adams F
Adams M
Addleman D https://orcid.org/0000-0002-6667-322X
Adnan A
Adroit B https://orcid.org/0000-0003-3693-9581
Afonso L
Aghajamali M
Ahmed J
Ahmed MR
Ahmed WH
Ahrens J
Alagarsamy T
Alahmadi A https://orcid.org/0000-0001-5359-1340
Alam M
Alessandretti L
Ali I https://orcid.org/0000-0002-6021-1411
Ali S
Aliannejadi M
Alizad-rahvar AR
Allen R
Alleyne M https://orcid.org/0000-0003-1049-6444
Alonso D https://orcid.org/0000-0002-8888-1644
Altgemissen J
Altmäe S
Altmann E https://orcid.org/0000-0002-1932-3710
Alzamly A
Ambike S
Ameen M https://orcid.org/0000-0002-2799-8988
Amos W https://orcid.org/0000-0002-0971-9914
Ancillotto L
Anderson J https://orcid.org/0000-0003-2441-0728
Angel-Sahagun C
Anikin A https://orcid.org/0000-0002-1250-8261
Anjanapura R
Ansah E https://orcid.org/0000-0003-2698-1452
Ansari D https://orcid.org/0000-0002-7625-618X
Antonioni A https://orcid.org/0000-0002-5788-3348
Anvari F https://orcid.org/0000-0002-5806-5654
Aoki N
Aramberri H https://orcid.org/0000-0003-2216-8931
Aren S https://orcid.org/0000-0003-1841-0270
Arévalo L https://orcid.org/0000-0002-0681-0392
Ariel C
Arnot M https://orcid.org/0000-0002-6293-5202
Arrigo F
Arrigo R
Arteaga O
Arumugam S
Asefa A https://orcid.org/0000-0002-6042-9592
Asghari-Kaljahi E
Assa H
Aste T https://orcid.org/0000-0002-4219-0215
Astolfi ML
Aston S
Attipoe A
Attwa M
Audet J-N
Auersperg A https://orcid.org/0000-0001-7405-9791
Auger-Méthé M
August A
Aune D
Avellan A https://orcid.org/0000-0001-6081-4389
Awasthi S https://orcid.org/0000-0001-8230-3635
Ayoub H https://orcid.org/0000-0003-3886-7323
Azadi S
B Gowtham https://orcid.org/0000-0002-8817-072X
Backman D
Badcock N
Bader T https://orcid.org/0000-0002-7829-4630
Baeten L
Bagley R
Bai Y
Baird E https://orcid.org/0000-0003-3625-3897
Baird T https://orcid.org/0000-0003-0052-8684
Bajardi P
Bajda T https://orcid.org/0000-0003-3849-5558
Bakker T
Balenzuela P
Ballard M
Baniel A
Banks-Leite C
Barclay P https://orcid.org/0000-0002-7905-9069
Barnard S
Barrafrem K https://orcid.org/0000-0003-3171-8640
Barreiro Felpeto A
Barrett P
Barron A https://orcid.org/0000-0002-8135-6628
Barton J
Barton P
Bastos A
Basu A
Basu J
Batailler C https://orcid.org/0000-0003-0553-6827
Bauer H
Bauer-Staeb C
Bayer YM https://orcid.org/0000-0002-3096-261X
Beare R
Beatty B https://orcid.org/0000-0002-5464-0041
Belal TS
Bell CG
Bellissent-Funel M-C
Belusic G https://orcid.org/0000-0003-3571-1948
Benítes A
Benitez H
Bennetts R
Bensch W
Bentley B
Benton C
Berens S
Bersacola H
Berthouze N
Bertolani R
Besancon L https://orcid.org/0000-0002-7207-1276
Bestelmeyer P
Betini G
Beveridge R
Bezuidenhout H
Bhandavat R
Bhaumik A
Bialek J
Bicca M
Biecek P
Biers T
Bilcik B
Billard L
Billeter J-C https://orcid.org/0000-0001-7796-4068
Binda P
Bindemann M
Biradar A
Birkedal H
Bishop D
Biswas G https://orcid.org/0000-0001-9097-6426
Biswas K
Bizhani M
Black A
Blackburn J https://orcid.org/0000-0003-0928-4831
Blackwood J https://orcid.org/0000-0003-2414-4722
Blain A
Blain B
Blalock L
Blazek R
Blettner M
Blöchl M
Bloom DD
Bloom S
Bochaton C https://orcid.org/0000-0003-4954-0019
Boddy C
Bode N https://orcid.org/0000-0003-0958-5191
Bode-Oke A https://orcid.org/0000-0002-2746-1822
Boersch-Supan P https://orcid.org/0000-0001-6723-6833
Boessenecker R https://orcid.org/0000-0002-6117-128X
Bolet A
Bonato M
Bor D
Borbás A
Borchers D
Borgman C
Borotikar B
Boruszewski P
Boteva L
Bottesini J https://orcid.org/0000-0002-5340-993X
Boucherie P
Bowler D https://orcid.org/0000-0002-7775-1668
Bracci S
Branch C https://orcid.org/0000-0003-1769-5040
Brange F
Branson R
Bretas R
Brick C https://orcid.org/0000-0002-7174-8193
Briefer E
Bright J
Brink E
Brizuela S
Brocki K
Brooks M
Brown G
Browne C
Browne N
Browning J
Brueningk S
Bruijn S https://orcid.org/0000-0003-0290-2131
Buchan S
Buck G
Buckingham G
Buescher J
Bulgariu L
Bullock J https://orcid.org/0000-0003-1023-3484
Bulman S
Buono Mónica
Burant J https://orcid.org/0000-0002-0713-3100
Burger R https://orcid.org/0000-0002-7361-3858
Burghardt G
Burmeister S https://orcid.org/0000-0001-5612-692X
Burridge H https://orcid.org/0000-0002-0719-355X
Buzna L
Bylapudi G https://orcid.org/0000-0002-5071-3557
Byrne B
Byrne H
Cade D https://orcid.org/0000-0003-3641-1242
Cahoon A
Cai Z
Caimi A
Calin-Jageman R
Calvo F
Campbell H
Campbell L
Cao M
Cao S https://orcid.org/0000-0001-9224-8887
Cao W
Cao Y https://orcid.org/0000-0002-9290-8504
Cao YP https://orcid.org/0000-0001-5976-2382
Capraro V https://orcid.org/0000-0002-0579-0166
Carabineiro SAC https://orcid.org/0000-0001-9913-4671
Carere C
Carey D
Carlos Moreno-Pirajan J
Carlson K
Carroll E
Carter M http://orcid.org/0000-0002-5351-8108
Casas J https://orcid.org/0000-0003-1666-295X
Cassiodoro G
Castagnola V
Castillo P
Castro GR
Catari G
Catlow R
Cavin L
Cecil K
Ceulemans R
Chakraborti A
Chakraborty D
Challands T https://orcid.org/0000-0002-7759-8966
Chamberlain R
Champagne C
Chander Y
Chandrasekar S https://orcid.org/0000-0003-2060-6029
Chang S-H
Chang Y
Chapman D https://orcid.org/0000-0002-3393-1261
Chaudhary J
Chaves Ó
Chazdon R
Chellasamy S https://orcid.org/0000-0002-1870-0377
Chen C https://orcid.org/0000-0002-5035-4021
Chen G https://orcid.org/0000-0002-6191-2906
Chen H
Chen J-T
Chen J
Chen J
Chen J https://orcid.org/0000-0001-9238-781X
Chen X https://orcid.org/0000-0002-9129-2197
Chen X https://orcid.org/0000-0001-9131-3798
Chen Y-C
Cheng K
Cheng Y
Cheung N https://orcid.org/0000-0003-1120-8926
Chikina M
Chira A https://orcid.org/0000-0002-2964-7583
Chityala PK
Choi SK
Choisne J https://orcid.org/0000-0001-5418-5870
Choonara Y
Chopik B
Chowdhury SN https://orcid.org/0000-0002-0326-826X
Chyba M
Cichota R
Clark A
Clark L
Clauss M https://orcid.org/0000-0003-3841-6207
Clement C
Clemente C https://orcid.org/0000-0001-8174-3890
Clifford S https://orcid.org/0000-0002-3774-3882
Close M
Coffin J
Cohen J
Colder CM https://orcid.org/0000-0003-0750-524X
Colin R
Collins S
Comas M
Combes F https://orcid.org/0000-0003-2658-7893
Conlan A https://orcid.org/0000-0002-2593-6353
Conner M
Cook MT
Coombs D https://orcid.org/0000-0002-8038-6278
Cooper C
Cooper R
Corballis M
Corcoran A https://orcid.org/0000-0003-1457-3689
Corcoran T
Cords M
Corker KS https://orcid.org/0000-0002-7971-1678
Corte L
Costa G https://orcid.org/0000-0002-6777-6706
Coulson S
Coutanche M https://orcid.org/0000-0002-2232-3519
Cox S https://orcid.org/0000-0002-9704-0716
Cramp R
Creel S
Cremer S
Cristea I https://orcid.org/0000-0002-9854-7076
Critchley H
Crouse J
Crowley B https://orcid.org/0000-0002-8462-6806
Crüwell S https://orcid.org/0000-0003-4178-5820
Cunha T
Curley J
Curnick D https://orcid.org/0000-0002-3093-1282
Curnow O https://orcid.org/0000-0001-7479-856X
Currat M
D'Août K
da Silva J https://orcid.org/0000-0001-5631-5421
Dahanukar N
Dahi-Taleghani A
Dai G http://orcid.org/0000-0001-9208-9994
Daley A
Dalton J
Damas Moreira I https://orcid.org/0000-0003-4630-3202
Damascena A
Daniels B https://orcid.org/0000-0002-5870-1059
Danulussi Alves Costa D https://orcid.org/0000-0002-1479-7677
Davey K
Davidson J http://orcid.org/0000-0001-9206-085X
Davies D
Davis RB
Davis T
Davy J
Dawber M
Dawson-Andoh B
Day AI
De Bastiani M
de Gelder B
de Hevia M-D
de la Madrid R
De Marco P
de Melo H
de Moraes LF
De Silva H
De Silva R https://orcid.org/0000-0003-0955-6366
De Tomi G
Deelchand D
del Valle L https://orcid.org/0000-0001-9916-1741
Delgado AV
Dellinger A
Deng X
Derex M https://orcid.org/0000-0002-1512-6496
Derocher A
DeVito N
Devoto M
Di Tommaso D
Diamanti MV
Dibari C
Dienes Z https://orcid.org/0000-0001-7454-3161
Dinesen G
Ding Y https://orcid.org/0000-0002-5953-3800
Dinis ML
DiRenzo G https://orcid.org/0000-0001-5264-4762
Dobney K https://orcid.org/0000-0001-9036-4681
Dodge S
Dominguez-Bello MG
Dong W
Donnellan B
Donner R
Dorgan K https://orcid.org/0000-0002-9687-9675
Dorozhkin S
Dorraki M https://orcid.org/0000-0002-6675-3393
Drabowicz J
Drax K https://orcid.org/0000-0002-2838-6211
Drenkova-Tuhtan A https://orcid.org/0000-0002-5163-7322
Drichoutis A
Driezen P https://orcid.org/0000-0003-2320-0999
Drobniak S
D'Souza A
Du J
Du T
Du X
Duan X
Dunderdale G
Dunleavy D https://orcid.org/0000-0002-3597-7714
Dunne J
Durak H
Durbeej B
Dutta A
Dutta D
Early C https://orcid.org/0000-0003-0308-2710
Ebach M
Eben C https://orcid.org/0000-0001-9423-1261
Ebrahimi A
Eckstein M
Edgington M https://orcid.org/0000-0003-1922-9529
Edmonds B
Eftimie R
Eissa MA
El Khatib N
El Moutaouakil A https://orcid.org/0000-0002-7407-020X
Elendner H
Elhattab A
Elmansi H https://orcid.org/0000-0002-3953-7169
Elyan E
Emam H
Enderling H
Endo A https://orcid.org/0000-0001-6377-7296
Engelhardt B https://orcid.org/0000-0002-8370-1317
Engelke S
Enticott G
Enweremadu CC
Eritja R
Ertekin E
Escobar LE https://orcid.org/0000-0001-5735-2750
Esquivel-Muelbert A
Esterhuyse S https://orcid.org/0000-0001-7675-443X
Estevez I https://orcid.org/0000-0002-9054-9528
Etchegaray C
Evans J https://orcid.org/0000-0001-9837-1026
Evans T https://orcid.org/0000-0002-6670-0718
Ewing L https://orcid.org/0000-0001-5263-1267
Ezcurra M https://orcid.org/0000-0002-6000-6450
Fadhil A
Faivre D https://orcid.org/0000-0001-6191-3389
Fan G
Fanelli D https://orcid.org/0000-0003-1780-1958
Fang L
Farina S
Farrar B
Farris D https://orcid.org/0000-0002-6720-1961
Farrugia N
Farzan R
Fathy ME
Fatima A
Favaro L https://orcid.org/0000-0002-8698-472X
Fedurek P https://orcid.org/0000-0002-6902-708X
Feigenson L
Feng G https://orcid.org/0000-0003-4828-5108
Feng J
Feng W
Feng Y
Fenske M
Ferguson V
Fernandes D
Fernandino L
Ferrante E https://orcid.org/0000-0002-2213-8356
Ferraz A https://orcid.org/0000-0002-5328-5471
Ferreri-Cancho R
Ferretti L
Field S https://orcid.org/0000-0001-7874-1261
Fiori M
Fischer J https://orcid.org/0000-0002-5807-0074
Fisher R https://orcid.org/0000-0002-2956-3240
Flammang B https://orcid.org/0000-0003-0049-965X
Flaxman S
Fleischmann P
Fleming S https://orcid.org/0000-0003-0233-4891
Fletcher-Watson S
Fluhr J
Fochler M
Foerder P
Forde T
Foster C https://orcid.org/0000-0003-1648-7485
Foster G
Fournet M https://orcid.org/0000-0001-7875-1857
Fox G
Franzoso G
Frasier T https://orcid.org/0000-0002-3055-0199
Frauendorf M https://orcid.org/0000-0003-1608-8396
Freedman-Fowler E
Freitas A
Fricke C http://orcid.org/0000-0002-0691-6779
Friston K https://orcid.org/0000-0001-7984-8909
Fromhage L https://orcid.org/0000-0001-5560-6673
Fry C
Frye M https://orcid.org/0000-0003-3277-3094
Fryer J https://orcid.org/0000-0002-3251-0860
Fuente C
Fujita K
Fuller A https://orcid.org/0000-0001-6370-8151
Furlong, L-A
Gagare S
Galán J https://orcid.org/0000-0003-3360-7602
Gallo D https://orcid.org/0000-0002-7409-7111
Gannett P
Gao H https://orcid.org/0000-0001-6852-9435
Gao J
Gao J
Garcia M https://orcid.org/0000-0003-2014-7387
García-Cano I
Garcia-Leon M
Garcia-Medina S
Gardner J
Garwood R
Gaudenzi R
Gauthier C
Ge H https://orcid.org/0000-0003-4659-7060
Gee B https://orcid.org/0000-0003-4517-3290
Gee C
Genwa K
Georgescu A
Georgopanos P
Gera R
Geraldeli S https://orcid.org/0000-0001-7837-5599
Gerlach M
Gerlee P
Gerth M https://orcid.org/0000-0001-7553-4072
Gheorghe D
Giese H
Gill P https://orcid.org/0000-0002-9020-4263
Gill R https://orcid.org/0000-0001-9389-1284
Gioia L
Giometto A
Girard-Buttoz C https://orcid.org/0000-0003-1742-4400
Glennerster A https://orcid.org/0000-0002-8674-2763
Gloag R https://orcid.org/0000-0002-2037-4267
Gluenz E https://orcid.org/0000-0003-4346-8896
Goetz C
Gómez-Rubio V
González-García F
Goodwin P
Gordon I
Gordon K
Graham T
Grange J
Grassi M
Gray K https://orcid.org/0000-0002-6071-4588
Grdadolnik J
Green A
Green KC https://orcid.org/0000-0001-9122-885X
Green M https://orcid.org/0000-0001-8978-9631
Gregorič M
Griette Q https://orcid.org/0000-0001-5978-9358
Grillo G
Gronau Q
Gross E
Gross T https://orcid.org/0000-0002-1356-6690
Grozinger C https://orcid.org/0000-0001-5738-1201
Gudla VC
Guerra LAA
Guess A
Guhr T
Gulbiński W https://orcid.org/0000-0001-9771-7451
Guo S https://orcid.org/0000-0002-5881-4942
Guo W https://orcid.org/0000-0003-3524-3953
Guo Y https://orcid.org/0000-0002-4985-7553
Guo Z
Gupta R
Gustison M
Guterstam A https://orcid.org/0000-0002-3694-1318
Haaf J
Haas W
Habanjar K
Hadfield-Menell D
Haensel J
Hagen S
Hagey T
Haifig I
Haines N
Hällfors M
Halliday W
Hämäläinen L
Hamamoto H
Hammerschmidt K
Hancean M-G https://orcid.org/0000-0001-9358-5287
Handa N
Handford M
Haneklaus N
Hanel P
Hanlon C
Haojun T
Haque M https://orcid.org/0000-0003-1836-8076
Harding H https://orcid.org/0000-0003-2511-3653
Hardwicke T https://orcid.org/0000-0001-9485-4952
Hardy J
Hariri-Ardebili MA
Harriman A
Harrison D
Harrison P
Harrison R
Harrison X https://orcid.org/0000-0002-2004-3601
Hartop E
Harvey N
Hasan MD
Hasshim N
Hatamoto M
Hayashi M
Hayward V
Hayyiratul F
He J
Heath S https://orcid.org/0000-0002-9822-8091
Heck PR
Hedge C
Heijungs R
Hein G https://orcid.org/0000-0001-5696-6486
Hejazi R
Hemery M
Henderson M https://orcid.org/0000-0002-2861-8668
Henebry GM
Henson R
Heo L
Herlinda S
Hermínio da Silva J
Hernandez SC
Hernandez-Vargas E https://orcid.org/0000-0002-3645-435X
Herrera J https://orcid.org/0000-0002-0633-0575
Herzel H
Heunis S
Hibbard P https://orcid.org/0000-0001-6133-6729
Higham D
Hilgard J
Hilgard J http://orcid.org/0000-0002-7278-4698
Hillaert J https://orcid.org/0000-0002-3761-8846
Hills T
Hirschi R
Hirt E
Hitzer E
Hobbs C https://orcid.org/0000-0002-2324-5882
Hochmann J-R
Hoekstra R https://orcid.org/0000-0002-1588-7527
Hoffrage U
Hohenberger A
Holland P https://orcid.org/0000-0003-1533-9376
Holmes I
Holowka N https://orcid.org/0000-0003-0593-7524
Honert E https://orcid.org/0000-0002-3865-5104
Honey M
Hong H
Hong J-W
Hood W https://orcid.org/0000-0002-8398-3908
Hopper G
Horikawa Y
Horváthová T
Horzum N
Hossain M
Hossain M
Hosseini SE
Hotchkiss F
Hou W https://orcid.org/0000-0001-7134-9558
Howes C
Hsiou A
Hsu N
Hu D https://orcid.org/0000-0002-0017-7303
Hu J
Hu Y
Hu Y
Hu Z
Hudgins E
Huffeldt N https://orcid.org/0000-0002-0154-2536
Hughes A https://orcid.org/0000-0003-2677-1965
Huijben S
Hunter A https://orcid.org/0000-0002-3527-5835
Hupman A
Husmeier D https://orcid.org/0000-0003-1673-7413
Hussin MH
Hutchins K https://orcid.org/0000-0001-8792-2830
Hyodo F
Iannelli F
IJzerman H https://orcid.org/0000-0002-0990-2276
Ilut¸iu V
Inagaki Y
Indra A
Ingham V
Innes N https://orcid.org/0000-0002-9984-0012
Invernizzi E
Iovannisci D
Iqbal A
Iqbal M https://orcid.org/0000-0002-9830-8235
Iribarrem A https://orcid.org/0000-0002-8540-9264
Isager P
Islam S
Itoh T
Ivanova VN
Ivanovska A https://orcid.org/0000-0001-6846-9583
Iwanicki T
Iwaniuk A https://orcid.org/0000-0001-9273-3655
Jackson B
Jacoby D https://orcid.org/0000-0003-2729-3811
Jaeger G
Jalili M https://orcid.org/0000-0002-0517-9420
Jamil A https://orcid.org/0000-0003-4947-2984
Jamshaid H
Janas M https://orcid.org/0000-0002-9206-8234
Janiga G
Janzen A https://orcid.org/0000-0002-6371-7469
Jarochowska E https://orcid.org/0000-0001-8937-9405
Jasuja K
Jaswal V
Jauch M
Jay T
Jejurikar S
Jell P
Jennings D https://orcid.org/0000-0001-5369-3574
Jha A https://orcid.org/0000-0003-3619-1788
Ji T
Jia L
Jiang R
Jiang X
Jiun YY
Johnson C https://orcid.org/0000-0002-9719-3771
Johnson S https://orcid.org/0000-0002-8648-1735
Johnson S
Johnston S https://orcid.org/0000-0003-4532-5573
Jones A
Jones B https://orcid.org/0000-0001-7777-0220
Jones S
Jordan N
José-Yacaman M
Joshi A
Joshi M
Joshi M
Jouiad M
Joyce D https://orcid.org/0000-0002-4253-5623
Juul JS https://orcid.org/0000-0002-5728-9269
Kaczka D
Kaklis K
Kalise D https://orcid.org/0000-0003-2327-1957
Kaltenberg A
Kankala R
Kanso E https://orcid.org/0000-0003-0336-585X
Kaňuch P
Kao R https://orcid.org/0000-0003-0919-6401
Kar P
Kara HE
Karakashev S
Karban R
Katz Y
Kaye L
Kazi T
Kelaher B
Keller A
Kellner A
Kelly L https://orcid.org/0000-0002-9736-0517
Kemp J https://orcid.org/0000-0003-3732-4992
Kharissov B https://orcid.org/0000-0001-7082-9357
Kibbe M
Kidd K
Kiefer M
Kim KJ
Kim TW
Kimmel M https://orcid.org/0000-0001-8161-890X
Kimura T
Kinateder M https://orcid.org/0000-0002-1669-3900
King G https://orcid.org/0000-0002-5327-7631
Kinouchi O
Kirilyuk V
Kirk R https://orcid.org/0000-0003-3739-2530
Klapiszewski Ł
Klauda JB
Kleinnijenhuis J
Kletskov A https://orcid.org/0000-0002-6979-545X
Kljajević LM
Knief U https://orcid.org/0000-0001-6959-3033
Knörnschild M https://orcid.org/0000-0003-0448-9600
Kobis N
Koenig U
Kohno Y
Kojima W https://orcid.org/0000-0003-2342-5944
Koks E
Kong N
Konstantinoudis G
Konstantinova T
Korb S
Korbel J
Korn C
Korošec M
Kostoglou M
Kotrschal A https://orcid.org/0000-0003-3473-1402
Kowshik M
Krajcsi A https://orcid.org/0000-0001-9792-9091
Kraus M
Kreegipuu K
Krishna A https://orcid.org/0000-0003-3838-9210
Krishnan K
Krishnan S http://orcid.org/0000-0002-6466-141X
Kristensen R
Kröncke I
Krout M https://orcid.org/0000-0002-0539-6986
Książek A
Kujala J
Kujala M
Kulahci I https://orcid.org/0000-0003-0104-0365
Kulkarni R
Kulshrestha J
Kumar Barui A
Kumar A
Kumar A https://orcid.org/0000-0002-6026-5727
Kumar A
Kumar A
Kumar G
Kumar N
Kumar S https://orcid.org/0000-0002-1066-3057
Kuperman M
Küpper F
Kurokawa S https://orcid.org/0000-0003-0560-6653
Kurt Z
Kwakkel J
Labuz J
Lacassagne L
Ladisch M
Laffoon JE
Lailvaux S
Laiou A
Laird AR
Lakens D https://orcid.org/0000-0002-0247-239X
Lakhno V https://orcid.org/0000-0001-9224-769X
Lam KF
Lambert O https://orcid.org/0000-0003-0740-5791
Lambertz C
Lammers M https://orcid.org/0000-0002-7215-4426
Lang M https://orcid.org/0000-0002-2231-1059
Larrouy-Maumus G
Latimer C https://orcid.org/0000-0003-0063-7506
Lau CH
Lau G https://orcid.org/0000-0002-0448-5071
Lau T https://orcid.org/0000-0002-0681-7295
Laurin M
Lavan N
Law S
Lawler R
Lawrence N
Lazar N
Lazrek HB
Le Lagadec R
Leakey R
Learmouth D
Leblanc R
Lebron Y https://orcid.org/0000-0001-6933-8454
Leclerc Q https://orcid.org/0000-0003-4761-001X
Lee CS
Lee O
Lehnert SJ
LEI CL
Lei F https://orcid.org/0000-0002-9169-8314
Lemaitre J-François https://orcid.org/0000-0001-9898-2353
Lemaitre J
Leng Y http://orcid.org/0000-0003-1685-1339
Leonenko V https://orcid.org/0000-0001-7070-6584
Leow M
Lesyk R
Lettieri M
Levens D
Levental I
Lewis P
Li A
Li B
Li C https://orcid.org/0000-0001-7516-3646
Li G
Li H
Li J
Li J https://orcid.org/0000-0003-2324-0501
Li K https://orcid.org/0000-0003-4864-3446
Li M
Li Q
Li S https://orcid.org/0000-0003-2575-6149
Li T
Li X
Li X-X https://orcid.org/0000-0002-3593-7536
Li Y
Li Z https://orcid.org/0000-0002-3230-5955
Liang M
Liang P
Liang X
Liao M
Lightman S
Lin D https://orcid.org/0000-0003-4492-0944
Liston J
Litchfield C
Little C
Liu C https://orcid.org/0000-0003-4168-8289
Liu F
Liu G
Liu H
Liu H
Liu J
Liu L
Liu Q
Liu XB
Liu X https://orcid.org/0000-0001-8574-3126
Liu X https://orcid.org/0000-0002-2299-9279
Liu Z
Liu Z
Loi F https://orcid.org/0000-0003-3045-3150
Lomberget T
Londhe V
Long RA
Lopez-Carmona MA https://orcid.org/0000-0001-9228-1863
Loued-Khenissi L https://orcid.org/0000-0003-3241-6492
Lourenço J
Lourenço M
Lovas-Kiss A
Loverdo C
Lovlie H https://orcid.org/0000-0003-4352-6275
Lu J https://orcid.org/0000-0002-5791-4749
Lucon-Xiccato T https://orcid.org/0000-0002-1017-9230
Lüpold S https://orcid.org/0000-0002-5069-1992
Lynch A
Lynch P
Ma A
Macdonald C https://orcid.org/0000-0003-2557-0520
MacLeod N https://orcid.org/0000-0002-7169-5036
Macnair W https://orcid.org/0000-0001-6357-0834
Macquarrie D
Madden J https://orcid.org/0000-0002-0691-0967
Madine J
Maggi R
Maharaj S
Maibaum T https://orcid.org/0000-0002-6627-5889
Maji VB
Makhija D
Malcolm P https://orcid.org/0000-0003-4110-4167
Maldonado M
Mallela J
Malod-Dognin N
Maminski M
Manassi M https://orcid.org/0000-0003-4210-7570
Manca L
Mani R https://orcid.org/0000-0002-2535-8987
Manickam R
Manousakas D
Mao X
Marble K
Marek Z
Marensi E
Mari L https://orcid.org/0000-0003-1326-9992
Mark-Lee WF https://orcid.org/0000-0003-3583-4163
Marks N
Marlair G
Márquez J
Marsh L
Marshall H https://orcid.org/0000-0003-2120-243X
Marshall N
Martin HG https://orcid.org/0000-0002-4556-9685
Martin-Duran J https://orcid.org/0000-0002-2572-1061
Martinez D
Martinez G https://orcid.org/0000-0002-7184-2569
Martinez-Felipe A
Martínez-García R https://orcid.org/0000-0003-2765-8147
Martinez-Lillo J
Martins LRM
Martins S
Martin-Torres J https://orcid.org/0000-0001-6479-2236
Martone P
Marzi S
Masli E
Mass GV
Massaro M https://orcid.org/0000-0001-8653-0472
Massazza A
Massen J
Massoud E
Mathger L http://orcid.org/0000-0002-0603-0345
Mathur M
Mathur R
Matre D
Matrosova VA
Matsuda H
Matsumi N
Maurits L
May J
Mayo M
Mazumder N
Mbare O
McAuliffe K https://orcid.org/0000-0002-4230-0250
McClure G
McCreesh N
McCulloch M https://orcid.org/0000-0002-3066-4989
McCune K https://orcid.org/0000-0003-0951-0827
McFarland R https://orcid.org/0000-0001-8245-9269
McGinty S https://orcid.org/0000-0002-2428-2669
McKamey S
McLoughlin S
McManus C https://orcid.org/0000-0003-3510-4814
McNeil J https://orcid.org/0000-0002-2702-0293
McPhaden M
Meese T https://orcid.org/0000-0003-3744-4679
Meléndez A https://orcid.org/0000-0002-5166-1840
Mello A
Menkhorst P
Menshykova M
Mercadante E
Metcalfe N https://orcid.org/0000-0002-1970-9349
Meyer B
Meylan P
Mia Z
Michael J https://orcid.org/0000-0003-4516-196X
Michalakopoulos T https://orcid.org/0000-0002-6944-2056
Michaud J https://orcid.org/0000-0001-6194-1355
Michilietto D
Midha P
Miksis-Olds J
Mikusinski G
Milfont TL
Milisavljevic D
Militao T https://orcid.org/0000-0002-2862-1592
Miller MF
Mills D
Mills D
Milward S
Minas G https://orcid.org/0000-0001-7953-706X
Mirande M
Mistry D
Mitchener W https://orcid.org/0000-0001-9687-1246
Mitra R
Miyano Y
Moazen M http://orcid.org/0000-0002-9951-2975
Moghadam M https://orcid.org/0000-0001-8984-1225
Moghaddam AZ
Mohamed A
Mohamed I
Mohammed IY
Mohan D
Monaghan P
Mondal A
Moneim A
Moog C https://orcid.org/0000-0003-3803-8760
Moon B
Moore S
Moors P https://orcid.org/0000-0002-4667-1235
Mørch CD https://orcid.org/0000-0001-6693-2028
Moreira VR
Morfin N
Morgan T
Morita M https://orcid.org/0000-0002-0170-1226
Morrison R https://orcid.org/0000-0001-9161-4734
Morrow I
Morton AB
Moseley P
Motallebzadeh H
Motta E https://orcid.org/0000-0001-9360-4353
Mousa ZM
Moustafa M https://orcid.org/0000-0002-1006-7685
Moysiuk J https://orcid.org/0000-0002-4685-5819
Muhammad A https://orcid.org/0000-0002-9017-2206
Mukherjee K
Muller E
Mullins P
Mumby P
Muneepeerakul R
Murdock D https://orcid.org/0000-0001-6838-5566
Muresan L
Muzzillo C
N, Prakash Prabhu
Nabavi E
Naghizadeh A
Nakamura T https://orcid.org/0000-0003-0183-1685
Nandiwale K
Naqinezhad A
Narwaz H
Nations J https://orcid.org/0000-0002-1170-7656
Naudet F
Navarro J
Nayga R
Nebelsick J
Nepal S
Ness R https://orcid.org/0000-0001-7313-8236
Newman J https://orcid.org/0000-0002-0815-7593
Newsome S https://orcid.org/0000-0002-4534-1242
Nguyen A
Nguyen H https://orcid.org/0000-0002-6496-7961
Nikoloski Z
Nilsonne G https://orcid.org/0000-0001-5273-0150
Nishimune H
Noakes P
Noh H-J https://orcid.org/0000-0003-4937-9093
Nollett C https://orcid.org/0000-0001-6676-4933
Nordqvist K https://orcid.org/0000-0001-8696-1707
Norman M https://orcid.org/0000-0002-1357-5415
Nosrati K https://orcid.org/0000-0001-9507-961X
Nouranian S https://orcid.org/0000-0002-8319-2786
Novas F
Novotny V
Nowacek D
Nuijten M
Nunney L https://orcid.org/0000-0002-4315-3694
Nuttall W
Nydam R https://orcid.org/0000-0002-2186-0216
Obidziński S
Obracaj D
O'Connor C
Oesch N
O'Hare A
Oji T https://orcid.org/0000-0002-1034-1111
Okabe T https://orcid.org/0000-0001-7518-5837
Okada I
Okamoto Y https://orcid.org/0000-0001-6511-4162
Olderbak S
Oldham S
Olivares F
Oliveira P
Oliveira-Filho F
Olivers C
Olson B
Önkal D
Opatowski L
Orben R https://orcid.org/0000-0002-0802-407X
Orian L
Orr T https://orcid.org/0000-0001-6247-5237
Ortega J
Ortega-Hernández J https://orcid.org/0000-0002-6801-7373
Osth A
Ouyang XK
Ovenden N
Owen D
Oyervides-Muñoz E
Ozbayram G
Ozgen G
Ozono H https://orcid.org/0000-0002-5495-9079
Padama A
Pagani L
Pagliara S https://orcid.org/0000-0001-9796-1956
Paiva V
Pal U https://orcid.org/0000-0003-2110-4610
Palm G
Palmer B
Pan M
Pan S https://orcid.org/0000-0001-9080-5651
Panagiotopoulou O https://orcid.org/0000-0002-6457-448X
Panaousis M
Pandolfi L
Pané, Salvador
Pang R
Panigrahi P https://orcid.org/0000-0003-1365-5252
Pant R
Papastamatiou Y https://orcid.org/0000-0002-6091-6841
Papastathopoulos Y
Parker D
Patil MS
Paulmann S
Paustian K
Pawbake A https://orcid.org/0000-0002-6345-489X
Pécheur E-I
Pellis S
Pelton JN
Peñalver E
Peng H https://orcid.org/0000-0001-6014-0249
Peng Z
Peredo C https://orcid.org/0000-0002-7217-9850
Pereira JM
Perna A https://orcid.org/0000-0001-8985-3920
Perrett D https://orcid.org/0000-0002-6025-0939
Peruzzo P
Peterka R
Peters E
Petit O
Pfalzner S
Phan N https://orcid.org/0000-0002-5928-7638
Photopoulou T https://orcid.org/0000-0001-9616-9940
Picardeau M
Pikovsky A https://orcid.org/0000-0001-9682-7122
Pilipović A
Pincebourde S
Pinto L
Pires A
Pisanski K https://orcid.org/0000-0003-0992-2477
Pissuwan D http://orcid.org/0000-0003-1244-7208
Plane J
Plohl N
Podos J
Pokern Y
Polajnar J
Polancyck R
Polet D https://orcid.org/0000-0002-8299-3434
Poli P
Pomeroy L
Pomi R
Ponchio C
Pongracz P https://orcid.org/0000-0001-5126-299X
Pope E https://orcid.org/0000-0001-5781-5575
Popkov V https://orcid.org/0000-0002-8450-4278
Portegies Zwart S
Pothuraju R
Powell L
Prabakaran S https://orcid.org/0000-0002-6527-1085
Pradillon F
Pratte Z
Preisser E https://orcid.org/0000-0002-8737-5619
Przybylski A https://orcid.org/0000-0001-5547-2185
Pugliese A
Pungetmongkol P
Purnell M
Pusch R
Qiu M
Quick N
Quintana M https://orcid.org/0000-0002-7036-8658
R. Scarano F
Rabi M
Racey P
Racsmány M
Rączaszek J
Radice V https://orcid.org/0000-0002-4867-0164
Radulescu O
Rageh A
RahJoo M
Rahman M https://orcid.org/0000-0003-2773-1244
Raisuddin S
Rajon E
Ramirez M
Ramírez-Argáez MA
Ramon M
Ramos J
Ramos M
Ranjbar A
Rao L
Rao R
Rauf A
Rawlence N https://orcid.org/0000-0001-6968-6643
Realmuto J
Rebuli N https://orcid.org/0000-0003-2339-974X
Recio G
Reese G
Reimann H https://orcid.org/0000-0002-3418-9345
Reisinger R https://orcid.org/0000-0002-8933-6875
Reisz R https://orcid.org/0000-0002-7454-1649
Reizner J
Rendell L https://orcid.org/0000-0002-1121-9142
Repnik R
Reshetnyak E https://orcid.org/0000-0002-9892-0459
Rey AR
Reynolds G
Reynolds J https://orcid.org/0000-0003-1536-1557
Ribas L
Ricci L
Rice A https://orcid.org/0000-0002-8598-9705
Richardson J
Richter M https://orcid.org/0000-0003-1868-9005
Riddle N https://orcid.org/0000-0003-1827-9145
Ridenhour B https://orcid.org/0000-0001-8271-4629
Riesch R https://orcid.org/0000-0002-0223-1254
Rigaud T https://orcid.org/0000-0002-0163-6639
Rindler A
Ringler C
Risch D
Risk MJ
Ritchie H https://orcid.org/0000-0001-9861-6644
Rizzo G
Robinson G
Rode K
Rodrigues A
Rodriguez N
Roemmich R
Rohenkohl G
Rohlfs M https://orcid.org/0000-0002-8767-1629
Rohrbeck C
Rohrer J
Rojas S
Rolim G https://orcid.org/0000-0002-8562-6189
Römer D https://orcid.org/0000-0002-7437-2195
Romero-Alvarez D
Rosa-Garcia A https://orcid.org/0000-0003-2223-007X
Rosipal R
Ross, SD
Rossberg A
Ross-Hellauer T
Ross-Marsh E
Roswell M
Rotella A
Roterman I https://orcid.org/0000-0003-3652-9099
Rothe J
Rounis E
Rovigatti L
Rowell T
Rowland M
Rubio-Fernandez P
Ruedi M https://orcid.org/0000-0003-3283-7764
Ruggera R
Rusconi E https://orcid.org/0000-0003-2700-205X
Ruvo M
Rychtar J https://orcid.org/0000-0001-6600-2939
Rydell J https://orcid.org/0000-0002-4930-6110
Ryu S https://orcid.org/0000-0001-9142-3030
Saberi M
Sacco I
Sachetto-Martins G
Sadhu A
Safari M
Safri A
Sahayaraj, K.
Sakala J
Sakthipandi K
Saldaña F https://orcid.org/0000-0003-0558-1169
Salerno G
Salmón P https://orcid.org/0000-0001-9718-6611
Samantaray P https://orcid.org/0000-0003-2533-929X
Samory M
Sanchez de Miguel A
Sanchez N https://orcid.org/0000-0002-6467-1781
Santamaria-Echart A
Santhanakrishnan A https://orcid.org/0000-0003-1800-8361
Santolamazza S
Santos FP
Sardanyes J https://orcid.org/0000-0001-7225-5158
Sargent J
Sarma SSS
Sartbaeva A https://orcid.org/0000-0003-1017-0161
Sathe B https://orcid.org/0000-0001-8989-0967
Saucedo O
Sauli P
Saunders F
Sawers A
Saxton T https://orcid.org/0000-0002-9902-5624
Saydam S
Sbricoli L https://orcid.org/0000-0003-2222-5764
Schaeffer P https://orcid.org/0000-0001-6230-8121
Scharkow M
Schenk H https://orcid.org/0000-0002-7427-2290
Schieber J
Schiffman J
Schleife A https://orcid.org/0000-0003-0496-8214
Schneck A https://orcid.org/0000-0001-7035-0346
Schneider CR
Schnell A https://orcid.org/0000-0001-9223-0724
Schöner M https://orcid.org/0000-0001-7538-7367
Schröder D
Schroeder R https://orcid.org/0000-0002-0613-3440
Schülke O https://orcid.org/0000-0003-0028-9425
Schurz H
Schuster S
Schwartz JJ
Schwen LO https://orcid.org/0000-0003-0195-9603
Schymanski S
Seale H
Seale M
Sedrez T https://orcid.org/0000-0003-3219-0729
Seeman S
Segalini A https://orcid.org/0000-0001-8667-0520
Seixas V
Semple S https://orcid.org/0000-0003-0452-8104
Sena E
Sengupta D
Sengupta D
Sequeira J
Serino A
Serino S
Serrano E
Sguotti C https://orcid.org/0000-0002-3019-6273
Shah S
Shang Q
Shao H
Sharbafi M https://orcid.org/0000-0001-5727-7527
Sharman J
Shawkey M https://orcid.org/0000-0002-5131-8209
Shelke N
Shen R
Shepherd A
Shi L
Shi S-Chen
Shimabukuro YE
Shimono H
Shinde S
Shingleton A
Shrestha N
Shrestha P
Shukla S
Siddique G
Sillen A
Sillero-Zubiri C
Silva B https://orcid.org/0000-0002-9870-2523
Silva R
Silveira Vitória R
Silverstein P
Silvestri S
Simmons R https://orcid.org/0000-0003-4964-8126
Simons M
Singh A
Singh R
Sinha S
Siripatana C
Sivasankar V
Skrabalak G
Skwarchuk S-Lynn
Slagter H
Smith D https://orcid.org/0000-0002-3427-0936
Smith KT
Smith S
Smithson M
Smotherman M
Sneddon L https://orcid.org/0000-0001-9787-3948
Soares M
Sobral G https://orcid.org/0000-0002-5001-4406
Soccol VT
Sogawa S https://orcid.org/0000-0003-2015-5707
Sogbanmu TO
Sohail M
Solazzo C https://orcid.org/0000-0001-5092-0807
Somers A https://orcid.org/0000-0002-0220-2904
Song G
Song S
Sonnenberg N
Sota T https://orcid.org/0000-0002-0400-9147
Souto C
Sparks A https://orcid.org/0000-0001-9916-2074
Speijer D https://orcid.org/0000-0002-2340-2753
Spies I
Spitzen J https://orcid.org/0000-0003-0455-7511
Spoelstra K https://orcid.org/0000-0001-8614-4387
Spritzer M
Srinivas K
Srivastava S
Srivathsa A https://orcid.org/0000-0003-2935-3857
Starostin E
Stefan A
Steinbauer M
Sterk AE
Stevanovic J
Sthel M
Stickland R https://orcid.org/0000-0003-3398-4272
Stienen E
Stoeger T
Stouffer D
Stovall A
Stramaglia S
Strandburg-Peshkin A https://orcid.org/0000-0003-2985-6788
Sturaro E
Stutt R https://orcid.org/0000-0002-1765-2633
Suc J-Pierre
Suckling, C
Sues H-Dieter
Sugai-Guérios M
Sugi Y
Suleman D. Shoaib
Sun B
Sutter A https://orcid.org/0000-0002-7764-3456
Suzuki M
Suzuki S
Svardal H
Swadzba-Kwasny G
Symonds M https://orcid.org/0000-0002-9785-6045
Számadó, Szabolcs https://orcid.org/0000-0003-2204-9705
Szczypinski F https://orcid.org/0000-0003-3174-8532
Szolnoki A
Szulkin M
Ta HT
Takei, T
Talbert P https://orcid.org/0000-0003-2563-3878
Tambouratzis T
Tang C-Jian
Tang D
Tang G
Tang J
Tanimoto J
Tarczay G
Tasca P
Taufer M https://orcid.org/0000-0002-0031-6377
Tay H
Tchuenche J https://orcid.org/0000-0001-8655-6154
Teixeira LSG
Teixeira S
Teletchea F
Teng Y
Teng Z
Tenreiro MJ https://orcid.org/0000-0003-4274-4879
Thibault R
Thomas A https://orcid.org/0000-0003-1239-883X
Thomas D https://orcid.org/0000-0003-2641-9304
Thompson J
Thompson N https://orcid.org/0000-0002-3195-1277
Thompson P
Thompson P
Thorogood R https://orcid.org/0000-0001-5010-2177
Thuy B
Tian S
Tilburg WAP https://orcid.org/0000-0002-9724-0603
Tindale S
Tinsley M
Tissier J https://orcid.org/0000-0002-8517-1612
Titz A https://orcid.org/0000-0001-7408-5084
Tiwari G
Toczyłowska-Mamińska R
Todd P
Toelch U https://orcid.org/0000-0002-8731-3530
Toivakka M
Tokita C https://orcid.org/0000-0002-8585-4317
Torezan JM
Torzewski J
Toyota K
Trail D
Tranos E https://orcid.org/0000-0002-9620-6542
Treede R-D
Trezise K
Tricase C
Trinajstic K
Trippier P
Tsaneva-Atanasova K
Tsang D
Tsoi L
Tsuihiji T
Tubiana L
Tulu B
Tung C-W https://orcid.org/0000-0001-5337-0177
Tung T-H
Turner N
Twomey K
Tzavella L
Unckless R https://orcid.org/0000-0001-8586-7137
V. K. J. Schmidt B
Vafidis A
Valdez G
Valencia-García R
Valen-Sendstad K
Vallianatos F https://orcid.org/0000-0002-4600-5013
Vámos T
van Aert R
van Baar J
van Kooten TG https://orcid.org/0000-0003-4953-8998
Van Parijs S
van Vugt M
van Zyl W
Vannucci A
Vargas A
Vargas-Bernal R https://orcid.org/0000-0003-4865-4575
Varnum M https://orcid.org/0000-0002-2088-6086
Vasconcelos M https://orcid.org/0000-0002-1215-0749
Vashista V
Vasquez K
Vasudev A
Verderane M
Vernes S https://orcid.org/0000-0003-0305-4584
Vernygora O
Verschuur J https://orcid.org/0000-0002-5277-4353
Vesely S
Viani R
Vicent JF
Vidavsky N
Vigne A
Villavicencio N
Villela D https://orcid.org/0000-0001-8371-2959
Vincent B
Vingerhoets G
Visscher D
Vivero RJ
Vladimirov N
Voinov P
Voller VR
Volodina E
Voltaggio M
Von reyn C
Vulic I
Waclaw B
Wada H
Wagner G https://orcid.org/0000-0003-1086-2301
Wakai S https://orcid.org/0000-0002-9400-4058
Walke J
Walker D
Wallot S
Walpole H
Walsh S
Waltert M
Wan Ismail WN
Wan K https://orcid.org/0000-0002-0291-328X
Wan Z https://orcid.org/0000-0003-2095-6336
Wang L https://orcid.org/0000-0002-5371-2138
Wang MC
Wang Q
Wang R
Wang S
Wang X https://orcid.org/0000-0002-5561-2763
Wang Y
Wang Y
Wang Y
Wang Z-X
Ward JA https://orcid.org/0000-0002-9637-6066
Watanabe H https://orcid.org/0000-0001-5031-9018
Waters J https://orcid.org/0000-0002-1514-7916
Wątorek M https://orcid.org/0000-0002-2131-7440
Watson D
Watson S
Watts D
Weary D https://orcid.org/0000-0002-0917-3982
Webb C
Weese D
Wei J
Weinstein N https://orcid.org/0000-0003-2200-6617
Welman J
Wen Z
West J
Westgate GE
Wheeler B https://orcid.org/0000-0002-8478-3385
Wheeler Q
Whitlock M
Whittow W
Widdig A
Wieczorek PP
Wiff R
Wiggins G https://orcid.org/0000-0002-1587-112X
Wijgerde T
Wild S https://orcid.org/0000-0002-5904-0096
Williams C
Williams J
Williams M
Wilson J
Wilson K http://orcid.org/0000-0001-5921-2145
Wilson S
Wilts B https://orcid.org/0000-0002-2727-7128
Wittmann M
Woehler E
Woerdenbag H https://orcid.org/0000-0002-9450-5880
Wolf C
Wolf L
Wondafrash M
Wong K-L
Wood J
Wood R https://orcid.org/0000-0002-1694-3295
Wooders M
Woodgate J
Wright A
Wright G
Wu A https://orcid.org/0000-0002-1801-5716
Wu H https://orcid.org/0000-0001-8869-6636
Wu J
Wu W
Wühr P https://orcid.org/0000-0003-3131-999X
Wyatt A https://orcid.org/0000-0002-1339-9546
Xiao C
Xiao C
Xiao T
Xiao W https://orcid.org/0000-0001-5245-4926
Xin L https://orcid.org/0000-0002-4683-4612
Xiong B
Xu C
Xu F https://orcid.org/0000-0001-9965-9646
Xu, JP
Xu L
Xu Z
Yagi S
Yamaguchi TT
Yamamoto S
Yamamoto T
Yan A https://orcid.org/0000-0001-7456-339X
Yan C https://orcid.org/0000-0002-5184-5391
Yan QL
Yan Y
Yang C
Yang H
Yang J https://orcid.org/0000-0001-5934-1537
Yang T-Y
Yang Y
Yannis G
Yawar A
Ye Y https://orcid.org/0000-0002-9278-9218
Yilmaz E
Yilmaz O
Yongchuan T
Young K https://orcid.org/0000-0002-1378-6415
Yu G
Yuan Y https://orcid.org/0000-0002-6698-3009
Yurkowski D https://orcid.org/0000-0003-2264-167X
Zaki E https://orcid.org/0000-0001-7053-3169
Zalucki J
Zanasi R
Zanchi C
Zanno L
Zanotti FL
Zanzotto FM
Zavodszky G https://orcid.org/0000-0003-0150-0229
Zayed S
Zekveld A https://orcid.org/0000-0003-1320-6908
Zeng X
Zhang B https://orcid.org/0000-0002-1617-6217
Zhang G
Zhang H https://orcid.org/0000-0003-1023-8007
Zhang L https://orcid.org/0000-0003-2431-869X
Zhang S-D
Zhang Y
Zhang Y
Zhang Z
Zhao C
Zhao J
Zhao Q
Zhao S
Zheng X https://orcid.org/0000-0001-6310-0162
Zheng Z
Zhiqiang F
Zhou G
Zhou H https://orcid.org/0000-0002-4210-6327
Zhou J
Zhou X
Zhuang B
Zibouche N
Zignani M
Zimova M https://orcid.org/0000-0002-8264-9879
Zurakowski R https://orcid.org/0000-0003-3726-3114
Zwaan R

